# Analysis of Expressed Sequence Tags of the Cyclically Parthenogenetic Rotifer *Brachionus plicatilis*

**DOI:** 10.1371/journal.pone.0000671

**Published:** 2007-08-01

**Authors:** Koushirou Suga, David Mark Welch, Yukari Tanaka, Yoshitaka Sakakura, Atsushi Hagiwara

**Affiliations:** 1 Nagasaki Industrial Promotion Foundation, Omura, Japan; 2 Josephine Bay Paul Center for Comparative Molecular Biology and Evolution, Marine Biological Laboratory, Woods Hole, Massachusetts, United States of America; 3 Faculty of Fisheries, Nagasaki University, Nagasaki, Japan; 4 Graduate School of Science and Technology, Nagasaki University, Nagasaki, Japan; University of Uppsala, Sweden

## Abstract

**Background:**

Rotifers are among the most common non-arthropod animals and are the most experimentally tractable members of the basal assemblage of metazoan phyla known as Gnathifera. The monogonont rotifer *Brachionus plicatilis* is a developing model system for ecotoxicology, aquatic ecology, cryptic speciation, and the evolution of sex, and is an important food source for finfish aquaculture. However, basic knowledge of the genome and transcriptome of any rotifer species has been lacking.

**Methodology/Principal Findings:**

We generated and partially sequenced a cDNA library from *B. plicatilis* and constructed a database of over 2300 expressed sequence tags corresponding to more than 450 transcripts. About 20% of the transcripts had no significant similarity to database sequences by BLAST; most of these contained open reading frames of significant length but few had recognized Pfam motifs. Sixteen transcripts accounted for 25% of the ESTs; four of these had no significant similarity to BLAST or Pfam databases. Putative up- and downstream untranslated regions are relatively short and AT rich. In contrast to bdelloid rotifers, there was no evidence of a conserved trans-spliced leader sequence among the transcripts and most genes were single-copy.

**Conclusions/Significance:**

Despite the small size of this EST project it revealed several important features of the rotifer transcriptome and of individual monogonont genes. Because there is little genomic data for Gnathifera, the transcripts we found with no known function may represent genes that are species-, class-, phylum- or even superphylum-specific; the fact that some are among the most highly expressed indicates their importance. The absence of trans-spliced leader exons in this monogonont species contrasts with their abundance in bdelloid rotifers and indicates that the presence of this phenomenon can vary at the subphylum level. Our EST database provides a relatively large quantity of transcript-level data for *B. plicatilis*, and more generally of rotifers and other gnathiferan phyla, and can be browsed and searched at gmod.mbl.edu.

## Introduction

Rotifera is one of the largest microinvertebrate phyla, in terms of both biomass and number of species. Its members are major components of freshwater and coastal marine ecosystems throughout the world, and are the chief non-arthropod component of most freshwater pelagic communities [1]. Most rotifers are smaller than 1 mm, but have ganglia; muscles; photo-, chemo-, and tactile sensory organs; structures for crawling, feeding, and swimming; digestive and secretory organs; and ovaries. The two major rotifer groups, Monogononta and Bdelloidea, are each significant for their unusual reproductive modes: bdelloids appear to be obligately asexual, reproducing only through mitotic division of germ cells; monogonont rotifers are facultatively sexual, generally reproducing through mitotic division of germ cells but entering a meiotic sexual phase in response to environmental cues [2].

The phylum is allied with other microinvertebrate groups such as Acanthocephala, Gnathostomulida, Micrognathozoa, and Cycliophora in the superphylum Gnathifera, a sister- or basal group to the Lophotrochozoa [3], [4]. Unlike other gnathiferan fauna, many rotifer species can easily be cultured in axenic conditions in quantities suitable for large-scale biochemical and molecular genetic studies, making rotifers the optimal model system for studying this basal animal assemblage. Among rotifers, the most well-studied are the euryhaline rotifers of the *Brachionus plicatilis* species complex. Their widespread distribution and ease of culturing make this group a useful model system for studies of population dynamics [5]–[9], speciation [10]–[14], the evolution of sexual reproduction [15], [16], and ecotoxicology [17]–[20]. The complex is also an important live food for the initial stage of larval rearing of marine fishes [21], [22]. However, while the biology, ecology, and culture conditions of many rotifer species have been studied, molecular and genetic studies are scarce and there are few genomic resources [10], [23]. Only genes used for phylogenetic analysis, such as those encoding ribosomal RNAs, cytochrome oxidase subunit I, and the 82kD heat shock protein have been widely sampled in Rotifera [11]–[14], [24], [25], and only a few others, such as those for the 70kD heat shock protein, a Mn-superoxide dismutase, and a ubiquitin-conjugating enzyme [26], [27], have been reported for *B. plicatilis*.

To increase the amount of genetic information available and to develop tools for further genomic research of rotifers, we constructed an unnormalized cDNA library from a mixed (asexual females, sexual females, and males) culture of *B. plicatilis*. We partially sequenced over 2300 clones and acquired data on the relative abundance, probable function, and sequence characteristics of more than 450 transcripts.

## Results

A rotifer culture is a complex microcosm of bacteria, protozoa, algae, fungi, and rotifers. A single rotifer may harbor as many as 10^8^ bacterial colony forming units (cfu) and a culture typically contains 10^3^–10^6^ bacterial cfu/ml [28]. The fungal species *Atkinsiella parasitica* was isolated from eggs and body of *B. plicatilis* [29], and PCR with primers to small subunit ribosomal RNA (SSU rRNA) demonstrated the presence of bacteria including *Afipia*, *Bosea*, and *Delftia*, protozoa including *Hartmannella*, and fungi including *Catenaria* in seemingly axenic cultures (DMW, unpublished). To create a cDNA library with minimal xenic contamination we established an axenic rotifer culture method (KS, unpublished). The food source, the algae *Chlorella vulgaris*, was cultured axenically. To minimize contamination of the library from *Chlorella* mRNA, rotifer culture at log phase growth was harvested aseptically, kept in sterilize seawater to allow the rotifers to consume remaining *Chlorella* and excrete all the food in their gut, and then washed several times with sterilize sea water. This procedure appears to have produced a cDNA library largely free of xenic contamination: none of the clones we sequenced had a top BLAST score to a fungus, and the gene encoding ribulose bisphosphatecarboxylase/oxygenase, one of the most expressed genes in plants [30]–[32], was absent.

**Figure 1:**
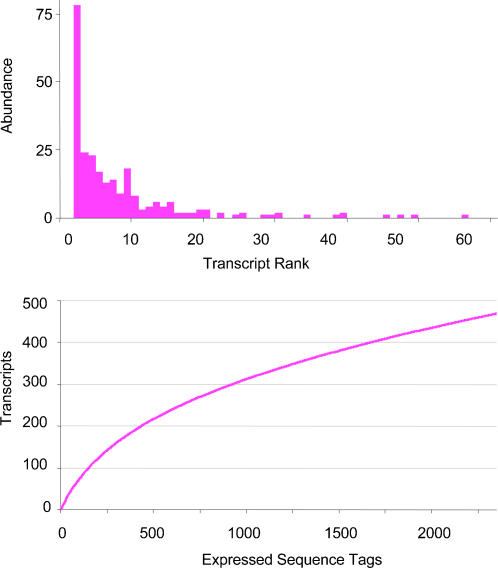
Transcript Abundance. a) Transcript abundance by EST abundance; the most abundant transcript (BpA0300 with 139 ESTs) is SSU rRNA and is not shown. (b) Rarefaction curve of transcripts predicted by number of ESTs.

After removing sequences that were vector-only, low quality, or polyA-only reads, 2362 ESTs were recovered with an average read length of 770 bases. Clones corresponding to 166 of the ESTs were subsequently sequenced in the opposite direction. Assembly output consisted of 534 contigs and 473 potential transcripts (Table 1). Ninety-nine of the contigs contained forward and reverse reads, 122 represented 61 supercontig scaffolds of transcripts sequenced in both directions with sequencing gaps, and 313 contigs represented transcripts sequenced in one direction only, of which 236 were composed of a single EST. Rank-abundance and rarefaction curves are shown in Figure 1. The redundancy of the library, calculated as 2362 ESTs/473 potential transcripts, was 4.99. The total number of transcripts present in the library was estimated to be about 800 using the Chao1 nonparametric estimator of total species richness [33], calculated as S_total_ = S_obs_+(S_1_^2^/2*S_2_) where S_1_ and S_2 _are the number of transcripts represented by 1 or 2 ESTs, respectively. Consensus contig sequences were deposited in DDBJ/EMBL/GenBank with accession numbers BJ979485 to BJ979996 and BJ999204 to BJ999251; individual ESTs were deposited in the dbEST division of GenBank with accession numbers ES466901-ES469274.

**Table 1:** cDNA Library and EST Database Properties

Mean insert size in kbSD (*n* = 40)	2.01.1
Recombinant efficiency	83%
Total reads	2678
ESTs after quality screening	2362
Total contigs	534
contigs with forward and reverse reads	99
contigs with forward reads only	435
supercontig scaffolds	61
contigs with one EST	236
supercontigs with 2 ESTs	19
Total inferred transcripts	473
rRNA transcrips	4
incompletely spliced transcripts	1
Total inferred coding genes surveyed	468
coding genes with 1 EST	232
coding genes with 2 ESTs	84

### Transcript function and abundance

Contigs were compared to nucleotide and amino acid sequences in GenBank and to Pfam whole domain and fragment HMM databases. Of the 473 transcripts comprising the 534 contigs, 371 had matches to BLAST databases with E values <1.0×e^−4^. Of the 103 transcripts that did not have significant similarity to BLAST databases, 53 had open reading frames of at least 300 bases and 22 had ORFs of at least 600 bases. However, only 12 had annotated Pfam motifs (as judged by an estwisedb bit score >25). The most abundant transcript was an SSU rRNA not eliminated in the mRNA purification (EST BpA0300); other transcripts with more than 1% of the total ESTs are listed in Table 2. In addition to ribosomal proteins, tubulin, and DNA binding proteins, these include transcripts encoding proteins similar to the cysteine protease Cathepsin L, a glutathione S-transferase, acyl-CoA oxidase, and a sphingolipid activator protein (saposin). Contigs representing four of the most abundant transcripts, each accounting for more than 1% of ESTs, have no significant BLAST or Pfam matches. The most abundant such transcript (BpA0604) is more than 1700 nt in length but lacks an ORF of significant length; it includes a tandem array of ten 68 nt repeats with an average pairwise identity of 95%. Another abundant transcript (BpA0292), composed of an ORF of at least 591 bases before a short 3′ UTR and a polyA tail, has no BLAST or Pfam similarity to database sequences, and is composed largely of serine-threonine repeats (none of the clones composing this contig contained an in-frame ATG near the 5′ end so the contig is probably an incomplete sample of the transcript).

**Table 2:** Highly abundant (>1%) non-SSU transcripts and their putative function. When two contigs make up a transcript scaffold both are listed; Score and E value are BLASTX to the nr database except for BpA0295 which is hmmpfam to Pfam_ls.

No. of ESTs (%)	Contig(s)	Acc. No.(s)	Gene product name or probable function	Score	E value
56 (2.5)	BpA0602	BJ999206	cathepsin L	406	8.00E-112
49 (2.2)	BpA0299	BJ979768	Peritrophin-A domain containing	53.6	5.90E-13
47 (2.1)	BpA0294	BJ979763	saposin-like	166	3.00E-39
45 (1.9)	BpA0604	BJ999208	no significant BLAST or Pfam similarities	—	—
39 (1.8)	BpA0276, BpA0288	BJ979746, BJ979757	DNA binding, R3H domain containing	96	1.00E-18
39 (1.8)	BpA0297	BJ979766	ribosomal protein L23	219	3.00E-56
38 (1.7)	BpA0296	BJ979765	Tubulin, beta, 2	493	0
34 (1.5)	BpA0295	BJ979764	no significant BLAST or Pfam similarities	—	—
30 (1.3)	BpA0284	BJ979753	no significant BLAST or Pfam similarities	—	—
30 (1.3)	BpA0285	BJ979754	Glutathione S-transferase	133	3.00E-30
29 (1.3)	BpA0601, BpA0610	BJ999205, BJ999214	DNA binding, similar to DEK oncogene	73.2	2.00E-22
28 (1.2)	BpA0292	BJ979761	no significant BLAST or Pfam similarities	—	—
25 (1.1)	BpA0291	BJ979760	acyl-CoA oxidase	221	4.00E-56
25 (1.1)	BpA0290	BJ979759	high-molecular-weight glutenin y-type subunit	55.8	1.00E-06

**Figure 2:**
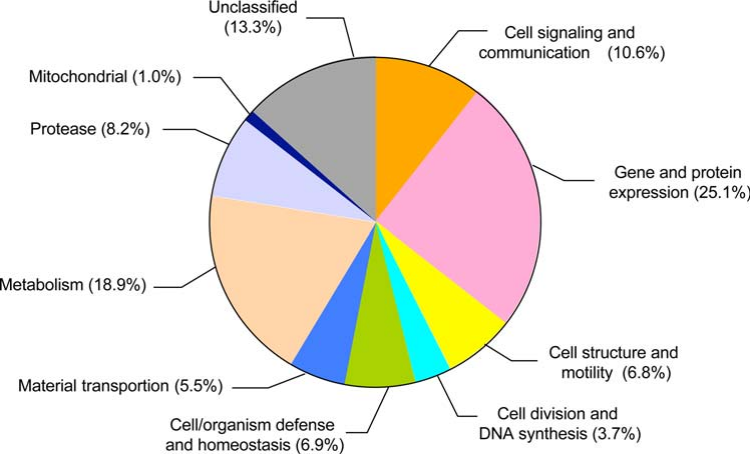
Transcript Abundance by Functional Class of Predicted Protein Product

Supplemental Table S1 lists all contigs in our EST database, the number of ESTs per contig, contig length, best BLAST hit, and the predicted functional category of the gene product when BLASTX to the nr database produced a match with a score >90 and E-value <1.0×e^−13^. In addition to the SSU rRNA gene sampled in BpA0300, single-EST contigs BpA0430 and BpA0435 contained different regions of 28S ribosomal gene(s) and single-EST contig BpA0464 contained a region of a 5S ribosomal gene. As shown in Figure 2, the categories with the greatest abundance of ESTs were gene/protein expression (25.1%), metabolism (18.9%), unclassified (13.3%), and cell signalling/cell communication (10.6%).

### Intragenomic comparisons

Every contig was compared to every other contig using BLASTN. One single pass read (BpA0008) was completely identical to another (BpA0417) except for a 351 base insertion. BpA0417 contains a complete ORF identified as ribosomal protein L11; BpA0008 has strong similarity to L11 by BLASTN, except for the 351 base insertion, which has no significant matches in BLAST or Pfam databases and does not contain an ORF of significant length. A genewise comparison of BpA0008 to the L11 protein of *Danio rerio* predicts an intron involving the insertion but not with exactly the same coordinates. The insertion begins with a GpT but does not end in an ApG and may represent an incorrectly spliced gene product. The only other contigs with significant similarity to each other were examined to confirm that they were assembled correctly; comparison of BLASTN hits showed these to be transcripts of different genes belonging to known gene families such as alpha- and beta- tubulins and low molecular weight heat shock proteins.

### Untranslated regions

**Figure 3:**
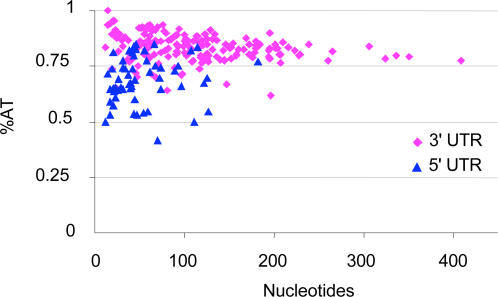
Distribution of UTRs by Length and %AT

The distribution of untranslated region (UTR) length and %AT is shown in Figure 3. The average length of the putative 5′ and 3′ UTRs was 52 bases (SD 35) and 94 bases (SD 74.5), respectively. The length of the putative 5′ UTR was not negatively correlated with overall transcript length, suggesting that the estimate of 5′ UTR length was not biased by incomplete first strand synthesis of the cDNA library (not shown). The 5′ UTR was examined for the presence of *trans*-spliced leader (TSL) exons [34] by cluster analysis, multiple sequence alignment, and all-to-all BLASTN comparisons. No sequence conservation indicative of a TSL exon was found.

The average %AT of the putative 5′ and 3′ UTRs was 68.7% (SD 10.3%) and 83.4% (SD 6.3%). The %AT of both UTRs is significantly greater than the overall %AT of the transcripts from which they were extracted (59.7% and 63.6%, respectively, by Student's T-test, p≪0.01). While there is great variation in the length of the putative 3′ UTRs, there is little variation in %AT. The 19 transcripts with putative 3′ UTRs greater than 200 bases in length have an average %AT of 81.1%, indicating that their base composition is similar to shorter 3′ UTRs and unlike the rest of the transcript, suggesting that they have been properly identified.

## Discussion

We obtained 2220 non-rRNA ESTs from our *B. plicatilis* cDNA library, which assembled into 530 unique contigs, 98 of which contained reads in forward and reverse orientation. A limited amount of bidirectional sequencing allowed us to determine that 122 contigs were forward and reverse pairs from a total of 61 transcripts. Some fraction of the remaining 310 contigs may also be forward and reverse pairs, particularly those containing a large and similar number of ESTs, but we have conservatively treated each as a separate transcript (with the exception of Bp0008, which we interpret as an incompletely spliced product of the same gene as Bp0417). The redundancy of the library (which decreases to 4.7 without the rRNA transcripts and incompletely spliced EST), is higher than libraries of other invertebrate metazoans, such as the copepod *Tigriopus japonicus* (2.62 [35]), the cladoceran *Daphnia magna* (2.44 [36]), the cnidarian *Cyanea capillata* (2.11[37]), and the nematode *Nippostrongylus brasiliensis* (1.66[38]). The reason for such high redundancy is unclear; however, we hypothesize that the expression of many genes may have been depressed during starvation. This is supported by the low estimation of total transcript diversity in the library estimated by rarefaction and by the Chao1 nonparametric estimator of total species richness, which both predict a total diversity of fewer than 1000 transcripts (Figure 1).

About 20% of the transcripts have no significant similarity to database sequences by BLAST, a lower fraction than in many EST studies, perhaps due to the starvation regime. Most of these transcripts contain identifiable ORFs but no recognized Pfam motifs, and represent genes of unknown function. Because there is little genomic data for Gnathifera the transcripts we found with no known function may represent genes that are species-, class-, phylum- or even superphylum-specific. These genes include four of the most abundant transcripts, which combined account for nearly 6% of the sequenced ESTs. Further study of these genes, of obvious importance to rotifers, will no doubt greatly expand our understanding of the biology and evolution of rotifers and other gnathiferans.

Although this library is far from complete, our database provides a large amount of information about the transcriptome of rotifers, including the predicted products of the highly expressed transcripts of *B. plicatilis* (Table 2). The most abundant non-SSU rRNA transcript encodes a cathepsin L-like cysteine peptidase. Cathepsin L has been identified in several arthropods, but the role of cathepsins in invertebrates is not known. The next most abundant transcript (BpA0299) contains a complete 1593 nt ORF with weak BLASTX matches (E>0.01) to insect chitinases and has three chitin binding Peritrophin-A domains identified by hmmpfam. In insects, proteins with these domains are associated with the peritrophic matrix lining the midgut and play a role in partitioning digestive enzymes, and in defence against ingested bacterial pathogens [39]. Both of these processes are important to understanding rotifer ecology and to aquaculture, and this gene provides a potential avenue to their study. Gamma-aminobutyric acid (GABA) has been shown to enhance population growth of *B. plicatilis* cultures, particularly in stressful conditions such as low food concentration and high free ammonia levels [40], [41], suggesting the existence of a GABA receptor and its importance for rotifer aquaculture. We found a transcript for a GABA receptor associated protein (BpA0418), which now allows the action of GABA in rotifers to be explored at the molecular level. Finally, one of our transcripts (BpA0140) has significant similarity to *trehalose-6-phosphate synthase* (*tps*) in BLAST and hmmpfam searches. This gene encodes the enzyme used in the first step of trehalose synthesis in most eukaryotes. The three ESTs that represent this transcript are similar to 5′ portion of *tps* containing the glycosyltransferase domain GT-20 (pfam00982, E value 2.6×e^−13^) associated with trehalose synthase activity, but the whole transcript has not been isolated. Trehalose has been detected in the resting eggs of *B. plicatilis* [42] but not in several bdelloid species [42], [43]. Unsuccessful PCR screens for *tps* in bdelloids have suggested that bdelloids are unable to synthesize trehalose [43]. Our discovery of a potential *tps* in *B. plicatilis* suggests that non-bdelloid rotifers use the *tps* pathway to synthesize trehalose and that bdelloids secondarily lost *tps* and the ability to synthesize trehalose.

Significantly, none of the genes we identified are related to the production of collagen, although collagens are very abundant proteins in most animals; other genes encoding proteins involved in cell structure, particularly laminin as well as actins, filamin, connectin, and tubulins, are present in our database. Biochemical assays have found no evidence of fibrous or non-fibrous collagens in *B. plicatilis* [44] and this is supported by the absence of collagen gene transcripts in our database. While acanthocephalans are known to contain collagen [45], the distribution of collagen in other rotifers and in other gnathiferan phyla is unknown. The presence of collagen genes may thus be a useful cladistic marker [4] and the morphological and physiological response over evolutionary time to the loss of collagen may be of interest.

We found no evidence of alternately spliced genes, although our survey was too small to rule out alternate splicing in rotifers. We found one transcript that appeared to be an incorrectly spliced version of another; if this interpretation is correct it would imply that the introns of the monogonont *B. plicatilis* are much larger than those found in several bdelloid rotifers (∼350 nt vs ∼60 nt, [46]; DMW unpublished). Other than genes belonging to known gene families (primarily alpha- and beta- tubulins) we found no evidence of multiple divergent gene copies in *B. plicatilis*, whereas this appears to be the norm in bdelloid rotifers ([47]; DMW unpublished). This provides additional evidence that the unique multiple-copy genome of bdelloids is related to their uniquely asexual evolution.

Another difference between monogonont and bdelloid rotifers apparent from our results is that monogonont rotifers appear to lack a conserved *trans*-spliced leader sequence. In contrast, bdelloid rotifers from two species belonging to different families were shown to have the same 23 nt exon sequence *trans*-spliced from ∼100 nt spliced leader (SL) RNAs to the 5′ end of more than 50% of their transcripts [48]. Searches of putative 5′ UTRs from our library revealed no similarity to the bdelloid SL exon, or any similarity among 5′ UTR sequences themselves. While our library was not enriched for 5′ ends, we did identify likely upstream regions in 68 transcripts. As our criteria were rather stringent, requiring identifiable similarity with the 5′ end of the coding sequence of a BLAST ortholog, this is no doubt an underestimate of the 5′ UTRs present in our database. If premature termination of first-strand synthesis had caused these 5′ UTRs to be incomplete, we would expect an inverse relationship between 5′ UTR length and total transcript length. No such relationship was observed, so most or perhaps all of the 5′UTRs we identified are probably full length. As TSL sequence was easily identified in more than 50% of bdelloid transcripts simply by comparing 5′ ends in a much smaller library using similar methodology, it seems likely that there is no conserved TSL sequence in *B. plicatilis*. However, we cannot exclude the possibility that only a small sub-set of low abundance transcripts are *trans*-spliced or that transcription of trans-spliced genes are suppressed under starvation conditions. If monogonont rotifers do in fact lack SL RNA, bdelloids and monogononts would be the most closely related groups that are known to differ in this phenomenon, and may thus provide a useful model system in which to study the evolution of SL RNA.

## Materials and Methods

### cDNA library construction and sequencing

*Brachionus plicatilis* Muller 1856 strain NH1L [49], a member of the *B. plicatilis sensu strictu* clade of the *B. plicatilis* species complex [11], was cultured at 25°C in Allen's artificial seawater [50] (diluted to 17 ppt salinity) inoculated with 3–7×10^6^ cells/ml *Chlorella vulgaris* strain K-73122 in a Jarfermentor (MBS, Japan). The algal food source *C. vulgaris* was cultured in bacteria-free medium, harvested by centrifugation, suspended in deionized, filtered water to a density of 8.6×10^9^ cells/ml supplemented with 800 ng/ml vitamin B12 (which is essential for rotifer growth [51], [52]) prior to introduction to the rotifer culture.

Rotifers were harvested by filtration onto a plankton net (45 micron mesh) that was washed with sterilized artificial seawater; rotifers were resuspended in 400 ml seawater that was exchanged every 2–3 hours over 12 hours to allow the rotifers to consume any remaining *Chlorella* and excrete their gut contents. The washed and starved rotifers were collected by plankton net and suspended in ISOGEN (Nippon Gene, Japan). The rotifers were homogenized with 0.8 mm glass beads using a vortex mixer and total RNA was isolated from the homogenate according to the manufacturer's instructions. Poly(A)^+^ RNA was purified from total RNA using MagExtractor (Toyobo, Japan). About 2.8 µg of poly(A)^+^ RNA was used to construct the cDNA library. Double-stranded cDNA was prepared using a cDNA Synthesis Kit (Amersham Bioscience, USA). The blunt-ended cDNA was ligated to the *Eco*RI/*Not*I adaptor, then ligated into *Eco*RI-predigested lambda-ZAPII arms (Stratagene, USA), and packaged *in vitro* by using Gigapack III gold packaging extract (Stratagene).

An aliquot of the cDNA library was incubated with *E. coli* XL1-Blue MRF to allow *in vivo* mass excision by ExAssist helper phage. The incubated solution was transfected into *E. coli* SOLR strain and plated on LB ampicillin plates containing X-Gal and IPTG to recover pBluescript SK (-) plasmids. Based on blue/white selection, the recombinant efficiency of the cDNA library was 83%. The average insert length from 40 randomly picked clones was estimated by restriction enzyme analysis to be approximately 2 kb (SD 1 kb).

Plasmid DNA was purified using a QIAGEN plasmid kit or a Genomic Solutions RevPrep robot, sequenced with ABI Big Dye 3.1 chemistry using standard M13 forward and reverse primers, and eluted on ABI PRISM 310, 3100, or 3730xl Genetic Analyzers (Applied Biosystems).

### Sequence analysis

All sequence data was examined independently using either DNASIS Pro software (Hitachi software, Japan) or unix shell scripts developed at the Josephine Bay Paul Center combining phred, phrap [53]-[55], lucy [56] trimseq, and trimest [57] and available from the corresponding author on request. Assemblies were compared and discrepancies examined and reconciled by hand. Contigs and unique sequences from singletons were compared to NCBI databases using BLASTN and BLASTX [58] and to Pfam databases using estgenewise [59] and hmmpfam [60]. Putative function was assigned based on best BLAST hit to a well-annotated sequence, and scored as similar if the top hit had a score greater than 50, and as highly similar if the top hit had a score greater than 90.

Sequences with significant similarity to database sequences by BLASTX were, when necessary, reoriented to match the subject sequence. All forward open reading frames greater than 150 bp were found, translated, and compared to databases by BLASTP. If an ORF began with an ATG, its set of top blast hits were similar to those found when blasting the entire transcript, and the ATG of the ORF was within 60 bases of the start site of at least one top blast hit, that ATG was considered the likely translation start site and all positions upstream to be part of the 5′ untranslated regions. Similarly, if a sequence contained a polyA tail and had an ORF with a set of top blast hits similar to those found when blasting the entire transcript, the stop codon of the ORF was considered the likely translation termination site, and all positions downstream but before the polyA tail were considered to be the 3' untranslated region.

## Supporting Information

Table S1 Table S1 is a tab deliminated ASCII text file containing Accession, # of ESTs, length, best BLAST hit information, predicted function, and predicted functional class for each contig.
(0.10 MB TXT)
